# Estradiol production of granulosa cells is unaffected by the physiological mix of nonesterified fatty acids in follicular fluid

**DOI:** 10.1016/j.jbc.2022.102477

**Published:** 2022-09-09

**Authors:** Vijay Simha Baddela, Marten Michaelis, Arpna Sharma, Christian Plinski, Torsten Viergutz, Jens Vanselow

**Affiliations:** Institute of Reproductive Biology, Research Institute for Farm Animal Biology (FBN), Dummerstorf, Germany

**Keywords:** NEFA, granulosa cells, estradiol, ERK1/2, gonadotropins, ALA, alpha-linolenic acid, BSA, bovine serum albumin, CYP19A1, cytochrome P450 family 19 subfamily A member 1, EGF, epidermal growth factor, FDR, false discovery rate, FA, fatty acid, FFA, free fatty acid, FOXL2, forkhead box protein L2, FSH, follicle-stimulating hormone, FSHR, Follicle Stimulating Hormone Receptor, GC, granulosa cell, IGF1, insulin-like growth factor 1, LA, linoleate, LH, luteinizing hormone, LSC, liquid scintillation counting, LHCGR, Luteinizing Hormone/Choriogonadotropin Receptor, NEFA, non-esterified fatty acid, OA, oleate, PA, Palmitate, RIA, radioimmunoassay, SA, stearate, SFAs, saturated fatty acids, USFs, unsaturated fatty acids

## Abstract

Ovarian cycle is controlled by circulating levels of the steroid hormone 17-β-estradiol, which is predominantly synthesized by the granulosa cells (GCs) of ovarian follicles. Our earlier studies showed that unsaturated fatty acids (USFs) downregulate and saturated fatty acids (SFAs) upregulate estradiol production in GCs. However, it was unclear whether pituitary gonadotropins induce accumulation of free fatty acids (FFAs) in the follicular fluid since follicle-stimulating hormone induces and luteinizing hormone inhibits estradiol production in the mammalian ovary. Interestingly, we show here the gas chromatography analysis of follicular fluid revealed no differential accumulation of FFAs between pre- and post-luteinizing hormone surge follicles. We therefore wondered how estradiol production is regulated in the physiological context, as USFs and SFAs are mutually present in the follicular fluid. We thus performed *in vitro* primary GC cultures with palmitate, palmitoleate, stearate, oleate, linoleate, and alpha-linolenate, representing >80% of the FFA fraction in the follicular fluid, and analyzed 62 different cell culture conditions to understand the regulation of estradiol biosynthesis under diverse FFA combinations. Our analyses showed co-supplementation of SFAs with USFs rescued estradiol production by restoring gonadotropin receptors and aromatase, antagonizing the inhibitory effects of USFs. Furthermore, transcriptome data of oleic acid–treated GCs indicated USFs induce the ERK and Akt signaling pathways. We show SFAs inhibit USF-induced ERK1/2 and Akt activation, wherein ERK1/2 acts as a negative regulator of estradiol synthesis. We propose SFAs are vital components of the follicular fluid, without which gonadotropin signaling and the ovarian cycle would probably be shattered by USFs.

Estradiol is a multifaceted sex steroid hormone essential for regulating the menstrual and estrous cycles in humans and animals. It acts as a growth hormone for tissues of the reproductive organs by supporting the development of the vaginal lining, glandular cervix, endometrium, myometrium, and lining of the fallopian tubules. Development of the mammary gland and maintenance of healthy bones, skin, liver, and brain also requires appropriate estradiol levels (1, 2). Ovarian granulosa cells (GCs) are the principal centers of estradiol production in females. During the reproductive cycle, pituitary follicle-stimulating hormone (FSH) and hepatic insulin-like growth factor 1 (IGF1) stimulate GCs' estradiol synthesis, which in turn triggers additional hypothalamic–pituitary events that are necessary for the pulsatile release of luteinizing hormone (LH) (3, 4). The LH surge commences the differentiation of estradiol-producing GCs into progesterone-producing luteal cells in the preovulatory follicle, followed by ovulation and the formation of the corpus luteum. In the luteal phase of the ovarian cycle, estradiol, together with progesterone, develops the endometrium to implant the early embryo (4). Therefore, regulation of estradiol synthesis in GCs is one of the critical events governing the ovarian cycle, fertility, and overall female health.

Palmitate (PA, C16:0), stearate (SA, C18:0), and oleate (OA, C18:1) are the predominant dietary fatty acids (FAs), which are also synthesized by the body's own metabolism in humans and animals. Consequently, these three FAs represent ∼70% of esterified fatty acids in adipose tissue (5) and nonesterified fatty acids (NEFAs) in circulation (6, 7). Under healthy conditions, free fatty acid (FFA) concentrations are lower in the blood as they are reserved in the form of stored lipids in adipose tissue. The systemic levels of PA, SA, and OA persistently rise in the blood during certain physiological stages (*e.g.*, fasting, negative energy balance, and lactation) and metabolic diseases (*e.g.*, obesity and diabetes). For more information on NEFA levels during metabolic diseases, please refer to our review article (3). Elevated levels of circulatory NEFAs cause a dramatic surge in their concentrations in ovarian follicular fluid (8, 9), plausibly *via* passive diffusion. Metabolic conditions/diseases characterized by increased concentrations of NEFAs are associated with female subfertility (3, 10, 11, 12, 13, 14). It has been implied that elevated levels of PA and SA are primarily responsible for the deterred fertility during metabolic diseases (15, 16). Contrary to this argument, our recent studies have shown that unsaturated fatty acids (USFs) decrease estradiol production, whereas saturated fatty acids (SFAs) increase the same. Similarly, a recent report by Zhou *et al.* (17) pointed out that OA induces downregulation of estradiol production in porcine GCs (17). However, since SFAs and USFs co-occur in the body fluids, the *in vivo* effects of NEFAs can only be better mimicked by culturing GCs in the presence of SFA and USF mixtures rather than by elucidating individual FA effects.

In the present study, we supplemented GCs with different combinations and concentrations primarily of PA, SA, OA, and alpha-linolenic acid (ALA, C18:3) to understand the regulation of estradiol synthesis. ALA is an omega-3 FA widely recommended for dietary intake to improve cardiovascular health. A growing number of publications suggest the beneficial role of ALA also on the reproductive health (18, 19). We have shown that ALA-rich diets could increase the levels of ALA in follicular fluid (20). ALA is not synthesized in humans and vertebrate animals such as cows and must be supplemented *via* diet to allow the synthesis of complex very-long-chain USFs and their derivatives. In addition to the above FAs, this study comprises two more USFs, palmitoleate (C16:1) and LA (linoleate, C18:2), which are also present in the follicular fluid. Therefore, the present study on different dietary and endogenously synthesized FAs helps to understand the physiological response of ovarian GCs to NEFAs under healthy and metabolic stress conditions.

## Results and discussion

### Follicular fluid NEFA levels are not affected by gonadotropins

**Figure 1:**
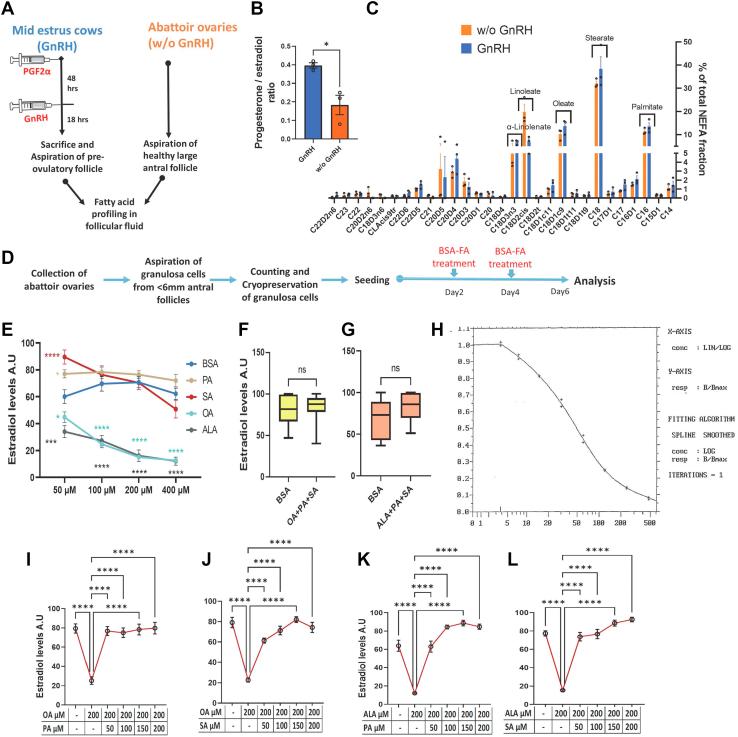
**NEFA species differentially regulate estradiol production.***A*, schematic representation of the follicular fluid collection strategy from GnRH-treated (n = 3) and GnRH-untreated abattoir (w/o GnRH) samples (n = 3) for fatty acid profiling. *B*, progesterone-to-estradiol ratio of follicular fluid samples used for fatty acid profiling. *C*, NEFA profiling data of GnRH-injected and GnRH-untreated (w/o GnRH) samples (n = 3). *D*, schematic representation of the procedure of primary granulosa cell culture. *E*, competitive radioimmunoassay measurements of estradiol production in the spent media of granulosa cells cultured under different fatty acid treatments at different concentrations (n = 8). *F*, estradiol production under combined treatment with 200 μM of OA, 100 μM of PA, and 100 μM of SA compared to BSA (n = 20). *G*, estradiol production under combined treatment with 200 μM of ALA, 100 μM of PA, and 100 μM of SA compared to BSA (n = 12). *H*, representative radioimmunoassay standard curve of estradiol spanning estradiol concentrations of 0 to 480 pg. The Y-axis indicates the B/Bmax values, while the X-axis indicates the concentrations (*I*) and (*J*) Estradiol production under different concentrations of PA and SA treated in combination with 200 μM of OA (n = 10). *K* and *L*, estradiol production under different concentrations of PA and SA treated in combination with 200 μM of ALA (n = 8). Probability values < 0.05 were considered as statistically significant and are designated with up to four asterisk symbols to inform about the strength of significant difference (∗*p* < 0.05; ∗∗*p* < 0.01; ∗∗∗*p* < 0.001, ∗∗∗∗*p* < 0.0001). n = indicates the number of independent cell culture replicates analyzed. ALA, alpha-linolenic acid; BSA, bovine serum albumin; FA, fatty acid; NEFA, nonesterified fatty acids; OA, oleate; PA, palmitate; SA, stearate.

It has been established that SFAs are lipotoxic while USFs such as OA and ALA are nontoxic and have favorable cellular functions (21, 22, 23, 24, 25, 26). However, our recent reports showed that OA and ALA downregulate estradiol production while PA and SA upregulate the same in GCs (27, 28), indicating the beneficial role of SFAs in female fertility and health. Since GCs' estradiol production is increased in the dominant follicle and decreased in the preovulatory follicle upon LH surge, we wondered whether there would be any distinct accumulation of FA species in the follicular fluid that can regulate the estradiol production during preovulatory follicle development. Therefore, we have analyzed the follicular fluid NEFA profile in GnRH-injected cows (post-LH staged) and compared it with control samples (pre-LH staged). Results revealed no significant differences in the levels of NEFA species between pre- and post-LH samples (Fig. 1*C*). SA was found to be the most abundant FA in the follicular fluid. PA, OA, LA, and ALA were the subsequent abundant FA species in both samples. Together, these five FAs contributed 79.18% and 78.36% of the total follicular fluids's NEFA fraction in pre- and post-LH staged follicular fluids. This analysis indicates that the FFA levels are not regulated by gonadotropins and remain unchanged in the follicular fluid during follicle maturation, meaning that follicular fluid contains a blend of different SFAs and USFs all the time. This is an important observation, which led us to wonder how GCs' steroidogenesis is regulated under such a mixed population of NEFA species, given the earlier knowledge that USFs downregulate and SFAs upregulate the estradiol synthesis.

### Dominant regulation of estradiol production by SFAs

Numerous studies in humans and animals implied that aberrant elevation of NEFA levels in follicular fluid during metabolic diseases is responsible for GC dysfunction (9, 29, 30, 31, 32). As mentioned in the introduction, we have primarily chosen PA, SA, OA, and ALA, which together make up the largest proportion of NEFAs in follicular fluid, to understand their cumulative effects on the regulation of estradiol production using the *in vitro* GC culture model. The schematic depiction of cell culture is mentioned in Figure 1*D*. Since FFA concentrations in the follicular fluid are highly variable among different physiopathological conditions (3, 8, 33), we first treated GCs with four different concentrations, spanning 50 μM, 100 μM, 200 μM, and 400 μM of PA, SA, OA, and ALA and compared the estradiol levels in the spent media to the corresponding concentrations of bovine serum albumin (BSA) vehicle–treated cells. Results showed that OA and ALA treatments resulted in a substantial and dose-dependent decrease in estradiol production (Fig. 1*E*). On the contrary, PA and SA supplementation increased the synthesis of estradiol at 50 μM concentration, but no significant changes were observed at other concentrations compared to the corresponding vehicle control. Despite higher estradiol concentrations compared to USFs, a trend of decrease in estradiol levels with increasing stearate concentrations was observed (Fig. 1*E*). Stearate is a direct substrate for oleate biosynthesis by the enzyme stearoyl CoA desaturase, which is abundantly expressed in GCs (34, 35). Therefore, the decrease of estradiol at higher stearate concentrations than its lower concentrations might be because of the increased biosynthesis of oleic acid from the exogenous stearate.

The dramatic downregulation of estradiol production by USFs eventually raises the possibility that USFs would affect the function of the dominant follicle by inhibiting the estradiol production, which is necessary for inducing the LH surge in different species, including humans. Since SA and PA are also abundant FAs in the follicular fluid in pre- and post-LH stage samples, we treated GCs with SFAs and USFs in different combinations and concentrations. To our surprise, the combination of 200 μM of OA or ALA together with PA (100 μM) and SA (100 μM) did not cause downregulation of estradiol production (Fig. 1, *F* and *G*). This suggests that SFAs overturn the USFs-induced downregulation of estradiol production in GCs. Figure 1*H* depicts the representative standard curve of the estradiol measurement using radioimmunoassay (RIA). To further understand this effect of SFAs, we performed biological titration experiments by supplementing GCs with 50 μM, 100 μM, 150 μM, and 200 μM of either PA or SA in combination with 200 μM of OA (Fig. 1, *I* and *J*). A similar set of experiments were conducted using 200 μM of ALA alongside different concentrations of SA and PA (Fig. 1, *K* and *L*). Results revealed a total reversal of OA- or ALA-induced downregulation of estradiol production upon PA or SA co-treatment at all subjected concentrations, indicating that GCs' estradiol production is upregulated and determined mainly by SFAs when a mix of SFA and USFs is present in the medium. Analysis of additional FA combinations using palmitoleate and LA has further confirmed the above-mentioned findings (Fig. S1). These results show for the first time that SFAs in the follicular fluid are highly efficient and indeed essential for overturning the inhibitory effects of USF on estradiol production by GCs.

### NEFA-induced regulation of gene expression

**Figure 2:**
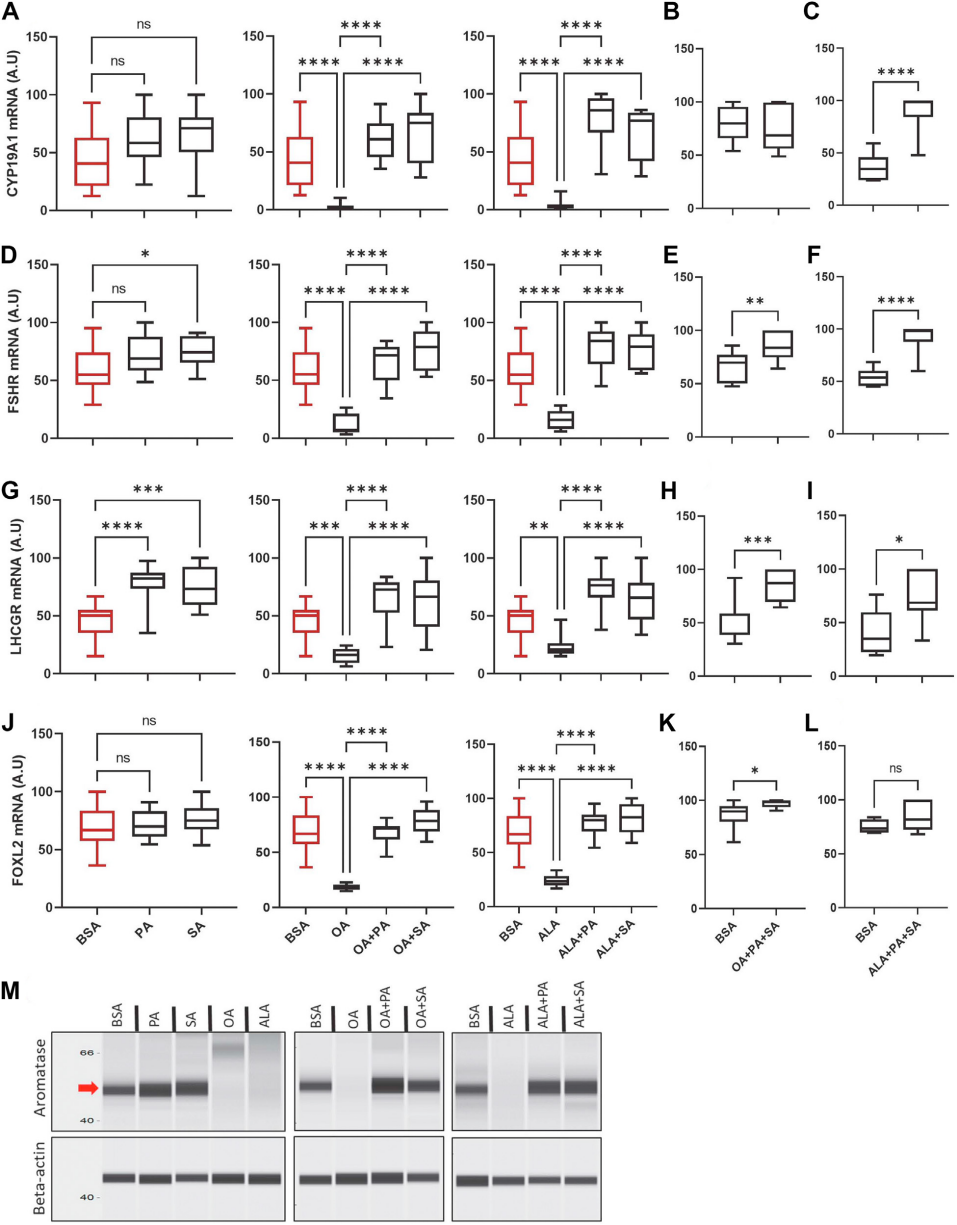
**Gene expression analysis.***A–C*, box plots of relative CYP19A1 mRNA abundance in granulosa cells derived from n = 12, n = 9, and n = 7 samples, respectively. *D–F*, box plots indicate relative FSHR mRNA abundance in granulosa cells derived from n = 12, n = 9, and n = 7 samples, respectively. *G–I*, box plots indicate relative LHCGR mRNA abundance in granulosa cells from n = 12, n = 9, and n = 7 samples, respectively. *J–L*, box plots indicate relative FOXL2 mRNA abundance in granulosa cells from n = 12, n = 9, and n = 7 samples, respectively. The BSA (vehicle) boxplot in the first three columns of the figure is identical since they are of the same combined experiment and hence placed under the same alphabetic identifiers (*A*), (*D*), (*G*), and (*J*) for clarification of the effects. *M*, digitally constructed images of aromatase and beta-actin proteins upon capillary electrophoresis of granulosa cell protein lysates collected on the sixth day of the cell culture. The left panel of the image indicates the molecular weight in kilodaltons. The *red* arrow indicates the aromatase protein band. Probability values < 0.05 were considered as statistically significant and are designated with up to four asterisk symbols to inform the strength of significant difference (∗*p* < 0.05; ∗∗*p* < 0.01; ∗∗∗*p* < 0.001, ∗∗∗∗*p* < 0.0001). n = indicates the number of independent cell culture replicates analyzed. BSA, bovine serum albumin; CYP19A1, cytochrome P450 family 19 subfamily A member 1; FSHR, follicle-stimulating hormone receptor; LHCGR, luteinizing hormone/choriogonadotropin receptor; FOXL2, forkhead box protein L2.

To determine the molecular regulation of estradiol production in GCs treated with different combinations of NEFAs, we performed expression analysis of different genes involved in estradiol production, such as CYP19A1 (cytochrome P450 family 19 subfamily A member 1), FSHR (follicle stimulating hormone receptor), LHCGR (luteinizing hormone/choriogonadotropin receptor), and FOXL2 (forkhead box protein L2). CYP19A1 encodes for the enzyme aromatase that converts androstenedione to estradiol in GCs. FSHR and LHCGR encode FSH and LH receptors, respectively, that induce follicular development and differentiation. The absence of FSHR and LHCGR severely compromises ovarian follicle development and ovulation, respectively (36, 37). FOXL2 encodes a forkhead transcription factor whose expression is essential for maintaining GCs' identity and thus ovarian function (38). Therefore, the expression of these genes, especially of CYP19A1, FSHR, and FOXL2, is crucial for maintaining estradiol production during follicular development. Results revealed that OA and ALA drastically decreased the expression of all these genes, while they were either increased or not affected by SA and PA (Fig. 2, *A–L*). Co-supplementation of either SA or PA or both to the OA- or ALA-treated cells prevented the USF-driven downregulation of gene expression (Fig. 2, *A–L*). These data indicate that NEFA-induced regulation of estradiol production occurs at the transcriptional level.

Importantly, these data suggest the dominant regulation of gene expression by SFAs over USFs in GCs. To clarify this further, we quantified the aromatase protein levels using the Wes instrument. Results revealed that aromatase protein levels are regulated in proportional to the CYP19A1 mRNA expression by FAs. OA- and ALA-treated cells did not show any traces of aromatase expression, while PA and SA treatments showed robust expression of the same (Fig. 2*M*). Further, as seen for mRNA, OA- and ALA-driven downregulation of aromatase protein expression is completely overturned in the presence of PA and SA (Fig. 2*M*). Quantification of aromatase protein data can be found in Figure S2 (supporting information 1). Together, these genes and protein expression data clearly indicate that SFAs in the follicular fluid are very essential for the proper regulation of the ovarian cycle by gonadotropins FSH and LH *via* corresponding receptors on GCs. Furthermore, these results also explain the underpinning mechanism of the data of Ferst *et al.* (39), where the bovine antral follicles were injected with a mixture of 200 μM PA, 200 μM SA, and 200 μM OA, and the follicular fluid along with the GCs were collected after 24 h. They found no differences in the expression of CYP19A1 and FSHR and the estradiol concentration between NEFA- and vehicle-injected animals (39), possibly due to the presence of SFAs in the injected NEFA mixture, as the present data reveal.

### Does NEFA-induced apoptotic cell death disturb the GC function?

**Figure 3:**
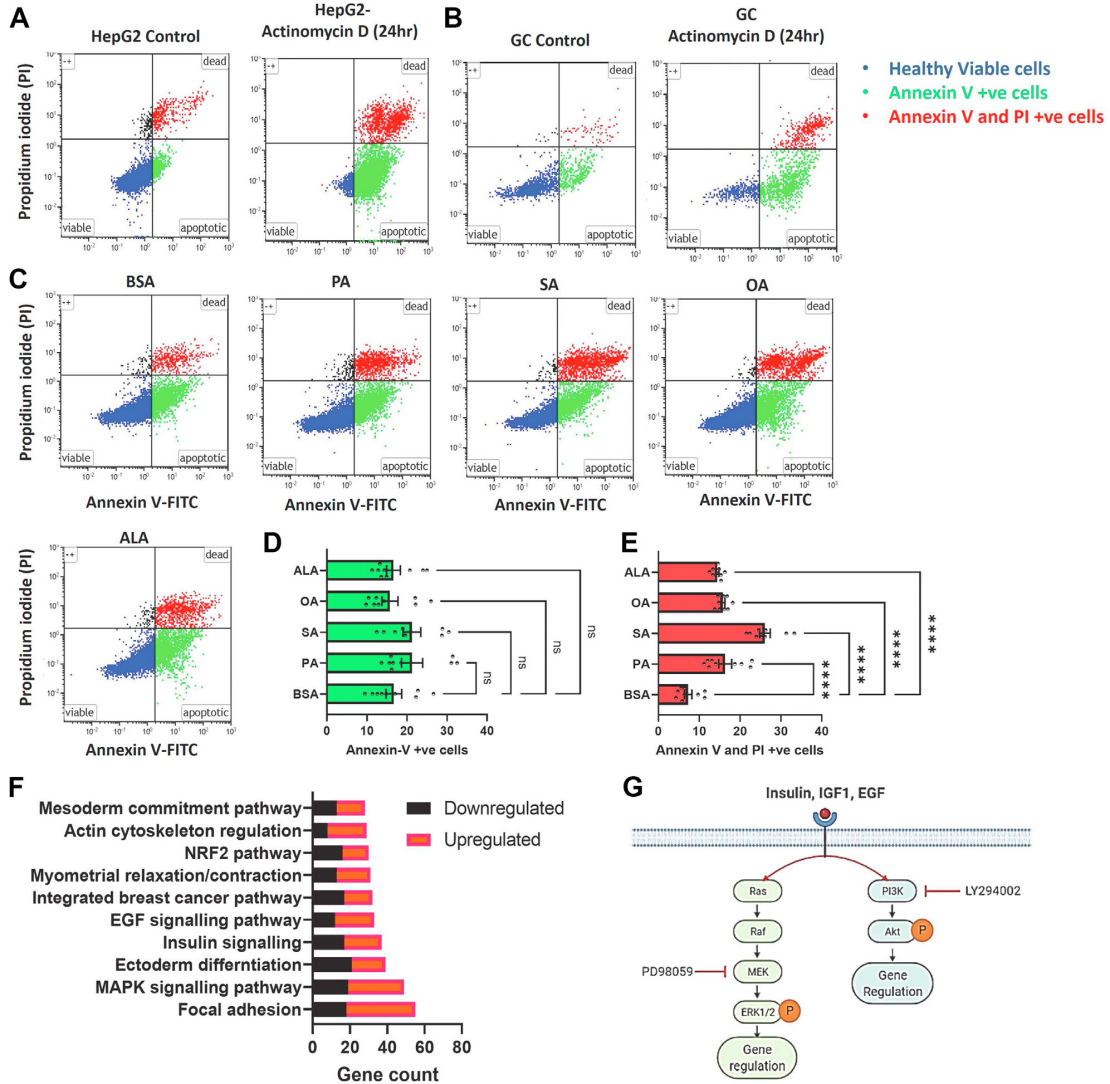
**Apoptosis and enriched pathway analysis.***A* and *B*, scatterplots of apoptosis analysis in cultured HepG2 and granulosa cells treated with or without actinomycin D. *C-E*the representative scatterplots of the early apoptotic cells (annexin V +ve), late apoptotic cells (annexin V +ve and PI +ve), and their cumulative data (n = 9). Probability values < 0.05 were considered statistically significant and are designated with up to four *asterisk* symbols to inform the strength of significant difference (∗∗∗∗*p* < 0.0001). *F*, highly enriched and significantly regulated pathways together with differentially regulated gene counts in oleic acid–treated granulosa cells. *G*, tyrosine kinase receptor signaling of insulin, IGF1, and EGF molecules and attack sites of inhibitors of the ERK1/2 (PD98059) and Akt (LY294002) pathways used in the present study. *G*, designed partly by using the BioRender available at app.biorender.com. n = indicates the number of independent cell culture replicates analyzed. EGF, epidermal growth factor; IGF1, insulin-like growth factor 1;

One of the crucial factors affecting estradiol production is the apoptotic status of GCs (40, 41, 42). Therefore, we asked whether the dramatic downregulation of estradiol production by USFs is due to increased apoptosis of GCs. Cultured HepG2 and GCs treated with or without actinomycin D (5 μM) for 24 h were employed as positive controls for apoptosis analysis. (Fig. 3, *A* and *B*). Flow cytometry analysis of annexin V and propidium iodide–stained cells revealed that FAs have no significant effect on the levels of early apoptotic cells (annexin V +ve and PI −ve) (*greens* in Fig. 3, *C* and *D*). In contrast, all FA treatments have significantly induced the proportion of late apoptotic cells (annexin V +ve and PI +ve) compared to BSA (*reds* in Fig. 3, *C* and *E*). The maximum amount (26.07%) of late apoptotic cells was observed in SA-treated cells, which plausibly resulted in the decreased estradiol levels at higher stearate concentrations seen in Figure 1*E*. At the same time, PA, OA, and ALA treatments resulted in approximately 15% GCs in the late apoptosis stage. The observed nonsignificant changes in the proportion of early apoptotic cells, unlike late apoptotic cells in FA treatments compared to BSA, could be due to the long-term cell culture model as depicted in Figure 1*D*. We supplemented cells with FAs for 4 days, which might have caused us to skip the detection of the early apoptotic cells, for which an earlier time point of analysis might have been beneficial. Since all FA treatments significantly induced apoptosis, we suggest that the NEFA-induced apoptotic cell death may be seen as normal due to increased cellular metabolism and cannot be attributed to the decreased estradiol production in USF-treated cells. These results on viability contradict the Mu *et al.* (43) report where they showed that 200 μM of PA- and SA-induced cell death by more than 50% in human GCs, whereas USFs did not affect the viability. However, it is important to acknowledge that Mu *et al.* (43) used 10% fetal calf serum for culturing the GCs and analyzed the numbers of viable cells *via* the trypan blue exclusion method using a hemocytometer (43) for data comparison. Importantly, ovarian follicles are devoid of direct blood supply; therefore, the inner follicular content, including follicular GCs, is not exposed to serum under physiological conditions. It has been reported that the addition of serum to cultured GCs nearly abolished estradiol production (44).

### ERK1/2 mediates the NEFA regulation of estradiol production

**Figure 4:**
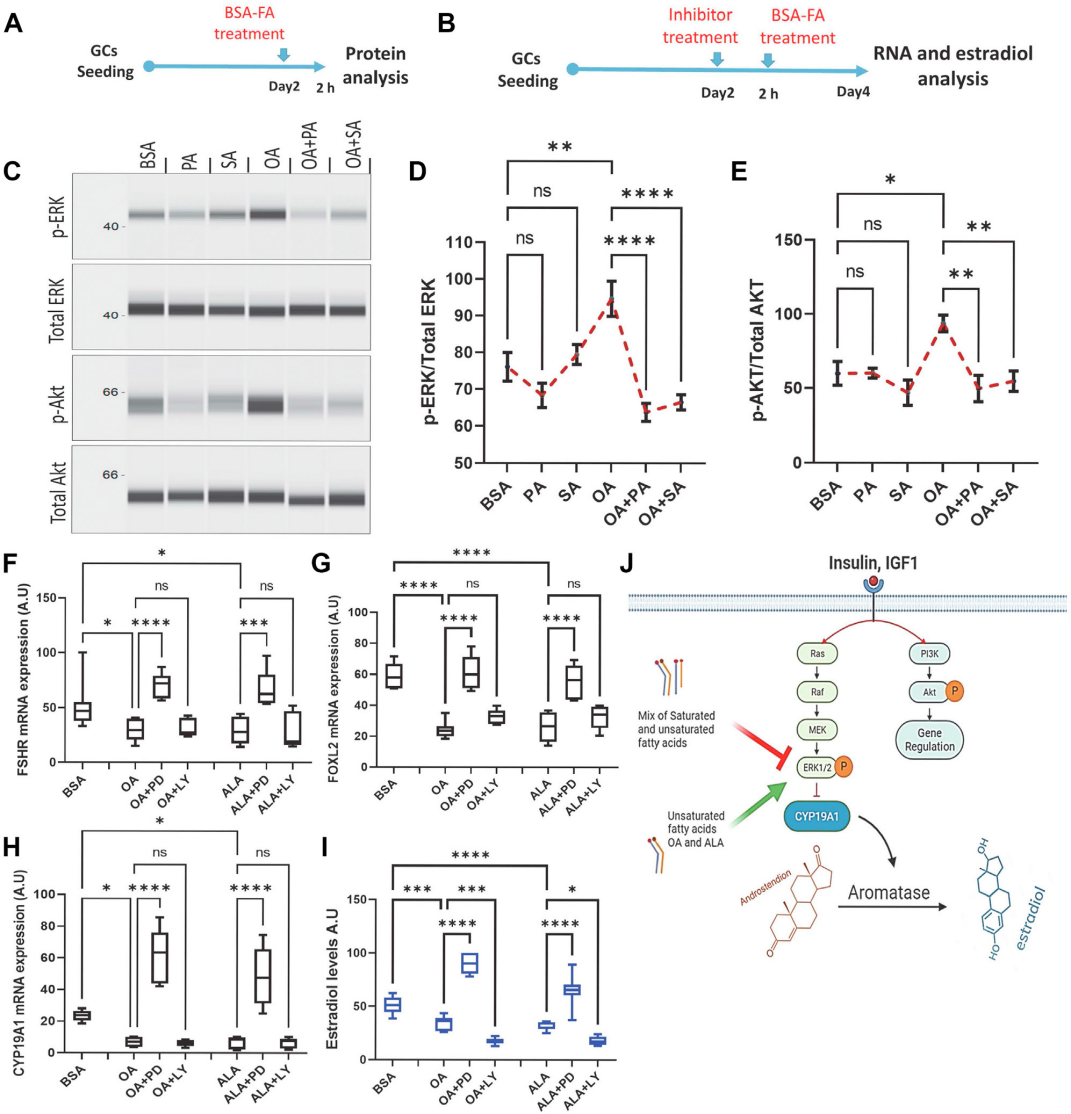
**ERK1/2 regulates fatty acid–induced estradiol production.***A* and *B*, schematic representations of cell culture procedures for phosphoprotein, gene expression, and hormone analysis. *C*, digitally constructed images of phospho- and total ERK1/2 and Akt proteins upon capillary electrophoresis of granulosa cell protein lysates collected after 2 h of fatty acid treatments. The left panel of the image indicates the molecular weight in kilodaltons. *D* and *E*, cumulative data of phosphoproteins/total proteins (n = 6). *F–H*, gene expression of FSHR, FOXL2, and CYP19A1 in OA- and ALA- treated granulosa cells in the presence or absence of inhibitors PD98059 (PD) and LY294002 (LY) (n = 7). *I*, competitive radioimmunoassay measurements of estradiol production in the spent media (n = 8). *J*, graphical conclusions of the present data: USFs such as OA and ALA promote ERK1/2 phosphorylation, thus inhibiting aromatase expression and estradiol production. In contrast, SFAs like PA and SA inhibit the same pathway, thus reversing the USFs-induced inhibition. *J*, designed partly by using the BioRender available at app.biorender.com. Probability values < 0.05 were considered as statistically significant and are designated with up to four asterisk symbols to inform the strength of the significant difference (∗*p* < 0.05; ∗∗*p* < 0.01; ∗∗∗*p* < 0.001, ∗∗∗∗*p* < 0.0001). n = indicates the number of independent cell culture replicates analyzed. ALA, alpha-linolenic acid; OA, oleate; PA, palmitate; SA, stearate; CYP19A1, cytochrome P450 family 19 subfamily A member 1; FSHR, follicle-stimulating hormone receptor; FOXL2, forkhead box protein L2.

We have recently reported a genome-wide transcriptome analysis of GCs treated with OA (GEO reference: GSE152307) (35). In the present study, we have reanalyzed the datasets and identified significantly regulated genes using false discovery rate (FDR) (q) <0.05 without considering the fold change cutoff value, which was previously used as a stringent selection criterion for identifying differentially regulated genes. This resulted in the identification of 2881 differentially expressed genes compared to the earlier 413 genes. Pathway enrichment analysis revealed that genes associated with map kinase (MAPK) signaling, insulin signaling, and epidermal growth factor (EGF) signaling are highly regulated by the OA treatment in GCs (Fig. 3*F*). It is well known that insulin, IGF1, and EGF receptors activate at least two different signaling pathways, such as protein kinase B (Akt) and ERK1/2 signaling *via* different intermediate molecules (Fig. 3*G*). Different published studies also support these bioinformatics interpretations as USFs such as arachidonic acid (AA) and OA were shown to induce ERK1/2 and Akt phosphorylation in GCs of different species (45, 46). In contrast, PA was reported to downregulate Akt phosphorylation in porcine GCs and KGN cells (31, 32) and induce Akt phosphorylation in skeletal muscle cells (47), indicating that different cell types respond differently to the FAs. Therefore, to determine how these signals are regulated in the present GCs, we analyzed the ERK1/2 and Akt phosphorylation levels under different combinations of FAs (for experimental design, see Fig. 4*A*). Results indicated that OA significantly induced phosphorylation of ERK and Akt proteins. Interestingly, co-treatment of PA or SA along with OA (Fig. 4, *C*–*E*) significantly decreased the phosphorylation of both these proteins compared to OA treatment, indicating that the counteracting effects of PA and SA on estradiol production may be mediated *via* inhibiting the activation of ERK and Akt signaling pathways by USFs.

To identify the role of these signaling pathways in estradiol biosynthesis, we pretreated GCs with chemical inhibitors LY294002 (LY) and PD98059 (PD), which are classified to specifically inhibit the Akt and ERK phosphorylation, respectively, for 2 h, followed by OA or ALA treatment for 48 h (for experimental design, see Fig. 4*B*). Analyses showed that PD treatment completely abolished the OA and ALA's inhibition of estradiol production and of the expression of FSHR, FOXL2, and CYP19A1 genes (Fig. 4, *F*–*I*). To further clarify the role of ERK in NEFA-induced estradiol production, we treated GCs with OA, OA+PD, and OA+PA+SA+PD and analyzed the estradiol levels. Results revealed that OA+PD– and PD+OA+PA+SA–treated GCs nearly phenocopy each other by producing similar levels of estradiol. Both treatments resulted in a significant increase in the estradiol levels compared to OA and thus confirmed the role of ERK in estradiol production (Fig. S3). These results are in line with the earlier findings where ERK1/2 signaling in human GC lines and mouse ovarian follicles was negatively correlated with estradiol production (48, 49). On the contrary, Mani *et al.* (50) showed that the inhibition of ERK1/2 signaling decreased the IGF1-induced estradiol production in bovine GCs. However, the CYP19A1 mRNA expression was not downregulated by the PD98059 treatment in that study (50).

Unlike ERK1/2, inhibition of Akt signaling using LY294002 did not reverse the OA- and ALA-induced downregulation of gene expression and estradiol production (Fig. 4, *F–I*). It was shown that Akt signaling in human cumulus GCs and bovine GCs induces GC steroidogenesis (50, 51). Contrary to these reports, our results clearly showed the downregulation of estradiol production in OA-treated cells despite high Akt phosphorylation. It has been shown that Akt hyperphosphorylation attenuates the downstream signaling molecules (52). Therefore, further investigations are needed to understand whether USFs induce AKT hyperactivation and, if so, how it impacts steroidogenesis in GCs. Although phosphorylation of both, Akt and ERK, was induced by OA and ALA, only inhibition of ERK, but not of Akt, could substantially abolish the negative effects of USFs on estradiol production and expression of corresponding key genes. However, further downregulation of estradiol production in OA-treated cells that were pretreated with LY294002 suggests an essential involvement of PI3k-Akt signaling in the basal level of estradiol production seen in OA-treated cells. These phosphorylation data suggest that ERK1/2 signaling could inhibit estradiol production despite high Akt phosphorylation. These data pave a strong basis for investigations into the differential effects of Akt and ERK1/2 signaling pathways and how their activation is regulated by different ligands (insulin, IGF-1, EGF, etc.) of tyrosine kinase receptors during dominant follicle development.

Since the estradiol synthesis of GCs was not affected by the coincident presence of saturated and unsaturated NEFAs, we wondered whether the implied NEFA-induced subfertility could be due to the reduced production of gonadotropin hormones such as FSH and LH, which are synthesized by the anterior pituitary gland and essential for the ovarian follicle development. An interesting study evaluating the effect of a high-fat diet on the pituitary expression of FSHB and LHB genes revealed no significant impact on the expression of these genes (protein expression data were not presented in the study), indicating NEFAs may not regulate the pituitary gonadotropin production (53). Alternatively, another critical component that could determine the regulation of the ovarian cycle is oocyte maturation and health. Since the present article is focused on estradiol production by GCs, we did not address the effects on the oocyte developmental competence in the present study and therefore suggest the need for further detailed evaluations of oocyte maturation to fully understand whether NEFAs (cumulatively) play a role in metabolic stress-/disease-induced ovarian dysfunction and subfertility in humans and animals.

## Conclusion

Taken together, the present data on steroidogenic GCs argue that the net effect of different NEFA mixes on estradiol production is neutral or positive but not negative. The cotreatment of either PA or SA or both to OA- or ALA-treated cells completely reverses the USF-driven inhibition of estradiol production by rescuing the gene expression of CYP19A1, FSHR, FOXL2, and LHCGR. Downregulation of estradiol production by USFs involved the induction of ERK1/2 signaling, which is reversed by PA and SA supplementation (Fig. 4*J*). Thus, SFAs in the follicular fluid are inevitable for rescuing the estradiol production from USFs and ensuring endocrine regulation of the ovarian cycle *via* gonadotropin receptors in GCs.

## Experimental procedures

### Follicular fluid FA profiling

Animal handling and follicular fluid collection were approved by the federal state of Mecklenburg Western-Pommerania, Germany. The follicular fluid preparation and gas chromatography analysis for FA profiling are performed as described earlier (28, 54). Two different groups of bovine follicular fluid samples were analyzed to determine whether there were any significant changes in the relative levels of FFA species. Among the two groups, one group of samples was collected from the dominant follicles of the ovaries derived from a local abattoir (DANISH CROWN Teterower Fleisch GmbH). The second group of follicular fluids was collected upon euthanization of PGF_2α_ (500 μg PGF Veyx forte, Veyx-Pharma GmbH) and gonadotropin-releasing hormone (100 μg GnRH analogue Gonavet Veyx, Depherelin; Veyx-Pharma GmbH)–injected animals (18 h later), which will have an LH surge 2 to 6 hours after the injection. The procedure of follicular fluid collection is schematically presented in Figure 1*A*. The morphological characteristics of the collected follicles are mentioned in Table S1 (supporting information 1). The increased progesterone-to-estradiol ratio in the GnRH-injected animals signifies that the collected follicles of these animals are in an early post-LH stage (Fig. 1*B*). The aspirated follicular fluid samples were immediately centrifuged at 3000*g* for 5 min to pellet the cells. The supernatant was collected and stored at −80 °C for gas chromatography analysis. Lipids were extracted from the follicular fluid using a chloroform–methanol mix (2:1, v/v) at room temperature. The FA profiling was performed using capillary gas chromatography with a 100 m × 0.25 mm CP-Sil 88 CB column (Chrompack-Varian) installed in a PerkinElmer gas chromatograph Autosys XL with a flame ionization detector and split injection (PerkinElmer, Inc).

### Isolation and culture of primary bovine ovarian GCs

Primary GCs collected from small- to medium-sized follicles were used for cell culture experiments (55). Ovaries were collected from the local abattoir (DANISH CROWN Teterower Fleisch GmbH) and transported to the laboratory in 1× phosphate buffer saline containing 100 IU/ml of penicillin, 100 μg/ml streptomycin, and 0.5 μg/μl amphotericin. GCs were harvested by manually aspirating the follicular fluid from <6-mm-sized ovarian follicles using a 3-ml syringe and 16G needle. Viable cells were counted *via* the trypan blue staining exclusion method and cryopreserved in freezing media (fetal calf serum containing 10% dimethyl sulfoxide). At the time of seeding of cells, cryovials were thawed in a water bath at 37 °C and washed using α-minimum essential medium (α-MEM). The cell pellet was dissolved in serum-free α-MEM containing 0.1% w/v FA-free BSA, 20 mM Hepes, 2 mM glutamine, 10 mM sodium bicarbonate, 4 ng/ml sodium selenite, 5 μg/ml transferrin, 10 ng/ml insulin, 1 mM nonessential amino acids, 100 IU/ml penicillin, and 0.1 mg/ml streptomycin. FSH (20 ng/ml), IGF1 (R3 IGF1, 50 ng/ml), and androstenedione (2 mM), purchased from Sigma-Aldrich, were added to the culture media before seeding. Cell culture dishes were coated with Collagen R (0.02%; SERVA Electrophoresis GmbH). Since FAs are physiologically complexed to albumin (56), all FAs were prepared by dissolving in FA-free 10% BSA solution to a stock of 5 mM concentration, containing FA-to-BSA molar ratio of 3.3 (57). Since SFAs are barely directly soluble in the BSA solution, they were initially dissolved in 2 ml of a chloroform–methanol solution (v/v = 2/3), which was later dried under nitrogen gas, followed by the addition of FA-free 10% BSA solution. The BSA–FA solutions were vortexed, placed in a water bath overnight, and stored in aliquots at −20 °C. GCs were cultured at a seeding density of 3.0 × 10^5^ cells/ml of the media. FA treatments were performed on day 2 and then on day 4 of the culture by replacing 70% of the spent media with fresh media containing FAs. On day 6, the spent media were collected for estradiol estimation, and cells were lysed for RNA and protein expression analysis depending on the experiment (Fig. 1*D*). To measure the phosphorylation status of Akt and ERK1/2 proteins, the cells were lysed after 2 h of FA treatment on day 2. For inhibitor studies, GCs were pretreated with 50 μM PD98059 and 50 μM LY294002 for 2 h on day 2, followed by the replacement of media with fresh media containing FAs. Cells were grown for 48 h before quantifying hormones and gene expression for inhibitor studies.

### RNA isolation, cDNA synthesis, and quantitative real-time PCR

RNA was isolated from the cultured cells using the innuPREP RNA Mini Kit (Analytik Jena). RNA concentration was quantified using a NanoDrop 1000 spectrophotometer (Thermo Fisher Scientific), and cDNA was synthesized using the SensiFAST cDNA synthesis kit (Bioline GmbH). SensiFAST SYBR No-ROX (Roche) reagent together with gene-specific primers (Table S2 of supporting information 1) were used for gene expression quantification. PCR products of each gene were initially cloned in the pGEM-T vector (Promega) and sequenced to verify the specificity of the primer pairs before analysis. Five different dilutions of cloned plasmids were used as standards and amplified together with cDNA samples in each run. The PCR amplification was performed in duplicates for each sample by taking 2.5 and 5 μl of cDNA in a total volume of 12 μl of reaction mix using a Light Cycler 96 instrument (Roche). PCR amplicon was verified for each run using melting curve analysis and agarose gel electrophoresis of PCR products.

### RIA quantification

The cell-free conditioned media at the end of the culture was collected and stored at −20 °C. For the assay, 5 μl of undiluted culture media were taken to measure 17-beta estradiol using the ultrasensitive competitive RIA. Tracer 2,4,6,7 -^3^H estradiol (Hartmann Analytic) was dissolved in 100% ethanol and diluted in RIA buffer. Estradiol antibody was custom generated by immunizing rabbits with estradiol conjugate and purified by chromatography (titer 1: 70,000). The estradiol standard (E-8875, Sigma-Aldrich) was dissolved in ethanol and diluted in PBS. The range of the estradiol standard curve was chosen from 480 pg/ml to 0 pg/ml *via* double dilution. The separation of free and antibody-bound estradiol was performed by the dextran-activated charcoal method. The levels of radioactivity were measured in a liquid scintillation counting system equipped with an integrated RIA-calculation program (TroCarb 2900 TR; PerkinElmer, Inc). The intra-assay and interassay coefficients of variation were recorded at 6.9% and 9.9%, respectively. All samples and standards were assayed in duplicates, the average of which was taken for the data analysis. Progesterone quantification was determined as reported earlier (57).

### Flow cytometry analysis of annexin V/propidium iodide staining

The cells were detached from the culture plate by incubating them with accutase (A6964, Sigma-Aldrich) for 20 min at 37 °C, transferred into 1.5 ml tubes, and washed with PBS by centrifugation at 300 × *g* for 5 min. Apoptotic status was then analyzed, according to the manufacturer’s protocol, by using the annexin V-FITC Kit (130–092–052, Miltenyi Biotec). The fluorescence signal was quantified from single cells (10,000 counts) using a flow cytometer (Gallios, Beckman-Coulter). The data were analyzed using the Kaluza software.

### Protein quantification by capillary electrophoresis

Protein expression analyses were performed by the capillary western method using the Wes instrument (ProteinSimple), according to the manufacturer's operational guidelines. Briefly, cultured cells in 48-well dishes were lysed using 50 μl of 1× MPER protein extraction buffer (Thermo Fisher Scientific), followed by centrifugation of the lysate at 12,000*g* for 3 min at 4 °C to collect the protein supernatant. Protein concentrations were determined using a micro-BCA protein estimation assay kit (Thermo Fisher Scientific). Protein samples, primary antibodies (1:50), wash buffers, blocking reagents, and chemiluminescent substrates were prepared and distributed into the predefined wells of the assay plates. The prepared assay plates were loaded onto the Wes instrument, and proteins were allowed to separate in a 12- to 230-kDa capillary separation module (SM-W001). Detection of protein bands alongside the molecular weight marker is an automated phenomenon of the instrument. The mice and rabbit primary antibodies used in the study are mentioned in Table S3 (supporting information 1). The anti-mouse (DM-002) and anti-rabbit (DM-001) secondary antibody modules were supplied by ProteinSimple company.

### Statistical analysis

Statistical analyses were carried out using the GraphPad Prism 9.0 licensed software. Data values were normalized for each experiment before performing statistical analysis. Multiple unpaired *t* tests were performed for the FA profiling data of GnRH-induced and abattoir samples. FDR (q values) of <0.1 were used as a threshold for recognizing significant changes in the FA data. Unpaired *t* tests were performed for analyzing the data of BSA *versus* OA+PA+SA– or ALA+PA+SA–treated samples. The estradiol data values in Figure 1*E* were analyzed using two-way ANOVA (concentration *versus* FA). The remaining data were analyzed using one-way ANOVA. Pairwise multiple comparisons were executed using post hoc tests. Data are presented as box plots with error bars spanning minimum to a maximum point of the data read or bar/scatter charts of mean with SEM values. Probability values < 0.05 were considered as statistically significant and are designated with up to four asterisk symbols to inform the strength of significant difference (∗*p* < 0.05; ∗∗*p* < 0.01; ∗∗∗*p* < 0.001; ∗∗∗∗*p* < 0.0001). The transcriptome data were analyzed using TAC 4.0 software (Affymetrix). Differentially expressed genes were identified using the FDR cutoff q <0.05. Significantly enriched pathways were identified using the inbuilt WikiPathways analysis (https://www.wikipathways.org) of the TAC 4.0 software.

## Data availability

All the data in support of the findings of this study are enclosed within the article and its supporting information.

## Supporting information

This article contains supporting information.

## Conflicts of interest

The authors declare no financial and nonfinancial competing/conflicting interests that could have appeared to influence the findings reported in the present article. All authors approved the article.
