# Supplementary material for: Estradiol production of granulosa cells is unaffected by the physiological mix of nonesterified fatty acids in follicular fluid
Source: J Biol Chem. 2022 Sep 9;298(10):102477. doi: 10.1016/j.jbc.2022.102477 (PMC9576879; doi:10.1016/j.jbc.2022.102477)
Supplement: Supplemental Tables S1–S3 and Figures S1–S3 [file mmc1.docx]

**Title:**

Estradiol production of granulosa cells is unaffected by the physiological mix of non-esterified fatty acids in follicular fluid

**Authors:**

Vijay Simha Baddela^1^, Marten Michaelis^1^, Arpna Sharma^1^, Christian Plinski^1^, Torsten Viergutz^1^, Jens Vanselow^1^

**Affiliations:**

^1^ Institute of Reproductive Biology, Research Institute for Farm Animal Biology (FBN), Wilhelm Stahl Allee 2, 18196 Dummerstorf, Germany.

**Correspondence:**

Vijay Simha Baddela, Institute of Reproductive Biology, Research Institute for Farm Animal Biology (FBN), Wilhelm-Stahl-Allee 2, 18196 Dummerstorf, Germany; Tel: +49 38208 68748; Fax: +49 38208 68752; e-mail: baddel@fbn-dummerstorf.de

**Short title:**

Regulation of estradiol production by NEFA

- **Supplementary Table 1:**

| S.No. | Follicle | Follicle Size (mm) | Follicular fluid Volume (ml) |
| --- | --- | --- | --- |
| 1 | GnRH 18 h sample 1 | 20 x 18 | 2.0 |
| 2 | GnRH 18 h sample 2 | 15 x 15 | 2.0 |
| 3 | GnRH 18 h sample 3 | 15 x 15 | 1.5 |
| 4 | Abattoir sample 1 | 15 x 18 | 2.0 |
| 5 | Abattoir sample 2 | 17 x 17 | 2.0 |
| 6 | Abattoir sample 3 | 12 x 15 | 2.0 |

Characteristics of the analyzed follicles for the follicular fluid fatty acid profiling

- **Supplementary Table 2:**

PCR primers:

| Gene | Sequence | Size (bp) | NCBI accession No. |
| --- | --- | --- | --- |
| **CYP19A1** | For. GCTTTTGGAAGTGCTGAACCCAAGG Rev: GGGCCCAATTCCCAGAAAGTAGCTG | 172 | NM_174305 |
| **FSHR** | For: TCACCAAGCTTCGAGTCATCCCAAA Rev: TCTGGAAGGCATCAGGGTCGATGTA | 189 | NM_174061 |
| **LHCGR** | For: GCATCCACAAGCTTCCAGATGTTACGA  Rev: GGGAAATCAGCGTTGTCCCATTGA | 205 | NM_174381 |
| **FOXL2** | For: AGCCAAGTTCCCGTTCTACG  Rev: GGTCCAGCGTCCAGTAGTTG | 140 | NM_001031750.1 |
| **RPLP0** | For: TGGTTACCCAACCGTCGCATCTGTA  Rev: CACAAAGGCAGATGGATCAGCCAAG | 142 | NM_001012682 |

- **Supplementary Table 3:**

Antibodies:

| Antibody | Product No | Brand | Source |
| --- | --- | --- | --- |
| Aromatase | SM2222P | Acris | Mouse |
| Phospho ERK1/2 | 9101S | Cell Signaling | rabbit |
| Total ERK1/2 | 4695S | Cell Signaling | rabbit |
| Phospho Akt (Ser473) | 9271S | Cell Signaling | rabbit |
| Total Akt | 9272S | Cell Signaling | rabbit |
| Beta Actin | SC47778 | Santa Cruz | Mouse |

- **Supplementary Figure 1:**

**
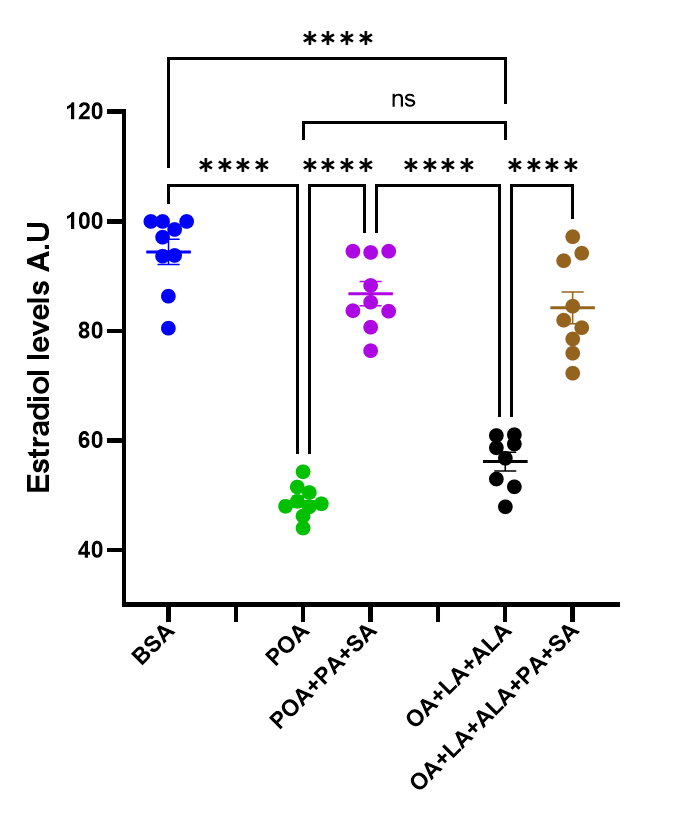
**

**Supplementary figure 1:** Estradiol production by granulosa cells upon treatment with different fatty acids. 200 µM of palmitoleic acid (POA) significantly decreased estradiol levels similar to other USFs like OA and ALA (See Fig 1). However, the co-supplementation of palmitate (100 µM) and stearate (100 µM) along with POA (200 µM) did not decrease the estradiol levels. Similarly, cells treated with a mix of three different unsaturated fatty acids oleate (100 µM), linoleate (100 µM), and alpha-linolenate (100 µM) decreased the estradiol production like that of OA or ALA, or POA-treated cells. Cosupplementation with palmitate (100 µM) and stearate (100 µM) along with OA, LA, and ALA did not decrease the estradiol levels. Cell culture and fatty acid treatments were given as per the scheme depicted in Figure 1D. **** = p < 0.0001. Scatter dots indicate the number of independent cell culture replicates analyzed.

- **Supplementary Figure 2:**

**
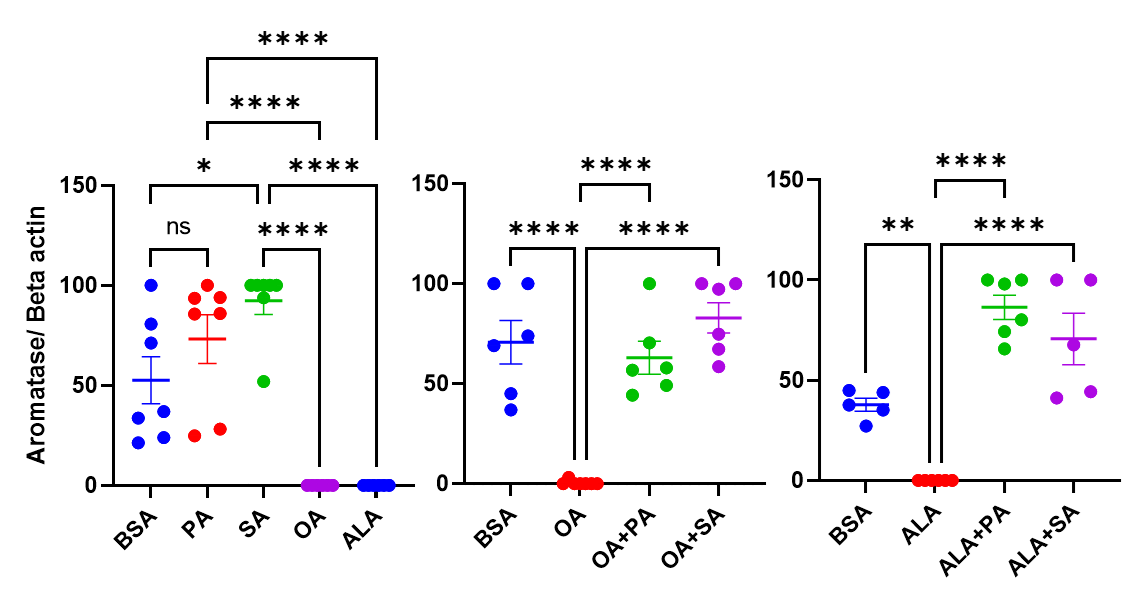
**

**Supplementary figure 2**. **Aromatase protein expression:** Relative aromatase protein levels in granulosa cells cultured in the presence of different fatty acids. Cell culture and fatty acid treatments were performed as given in the scheme depicted in Figure 1D. Scatter dots indicate the number of independent cell culture replicates analyzed.

- **Supplementary Figure 3:**

*
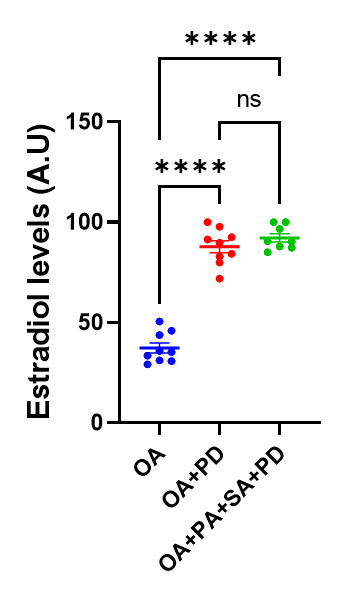
*

**Supplementary figure 3**: Estradiol production by granulosa cells upon treating OA, OA+PD, and OA+PA+SA+PD. Inhibitor treatments were given as per the cell culture scheme depicted in Figure 4B. **** = p < 0.0001. Scatter dots indicate the number of independent cell culture replicates analyzed.
